# Two-Exposure Image Fusion Based on Optimized Adaptive Gamma Correction

**DOI:** 10.3390/s22010024

**Published:** 2021-12-22

**Authors:** Yan-Tsung Peng, He-Hao Liao, Ching-Fu Chen

**Affiliations:** Department of Computer Science, National Chengchi University, Taipei City 116, Taiwan; 110753115@nccu.edu.tw (H.-H.L.); ecpoem@gmail.com (C.-F.C.)

**Keywords:** two-exposure image fusion, gamma correction, high dynamic imaging

## Abstract

In contrast to conventional digital images, high-dynamic-range (HDR) images have a broader range of intensity between the darkest and brightest regions to capture more details in a scene. Such images are produced by fusing images with different exposure values (EVs) for the same scene. Most existing multi-scale exposure fusion (MEF) algorithms assume that the input images are multi-exposed with small EV intervals. However, thanks to emerging spatially multiplexed exposure technology that can capture an image pair of short and long exposure simultaneously, it is essential to deal with two-exposure image fusion. To bring out more well-exposed contents, we generate a more helpful intermediate virtual image for fusion using the proposed Optimized Adaptive Gamma Correction (OAGC) to have better contrast, saturation, and well-exposedness. Fusing the input images with the enhanced virtual image works well even though both inputs are underexposed or overexposed, which other state-of-the-art fusion methods could not handle. The experimental results show that our method performs favorably against other state-of-the-art image fusion methods in generating high-quality fusion results.

## 1. Introduction

**Figure 1:**
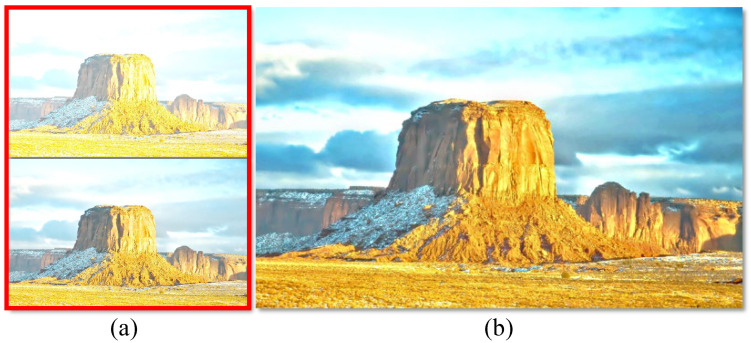
An example of the proposed two-exposure image fusion. (**a**) An input image pair with two exposures. (**b**) The fused image using the proposed method.

Image fusion has been a crucial low-level image processing task for various applications, such as multi-spectrum image fusion [1,2], multi-focus image fusion [3], multi-modal image fusion [4], and multi-exposure image fusion [5]. Among these applications, thanks to smartphones’ prevalence with their built-in cameras, multi-exposure image fusion is one of the most common applications. Since most natural scenes have a larger ratio of light to dark than what a single camera shot can capture, a single-shot image usually cannot present details of high dynamic ranges, thus having under- or overexposed parts for the scene. When a camera captures an image, its sensors can only catch a limited luminance range during a specific exposure time, resulting in a so-called low-dynamic-range image. An image taken for short exposure tends to be dark, while it is bright for long exposure, as shown in Figure 1a. Fusing differently exposed low-dynamic-range (LDR) images to obtain a high-dynamic-range (HDR) image requires extracting well-exposed (highlighted) regions from each LDR image to generate an excellent fused image, which has been very challenging.

Several research works have been performed for Multi-scale Exposure Fusion (MEF) [6,7,8]. In general, it is common to fuse LDR images using a weighted sum, where the weight associated with each input LDR is determined in a pixel-wise fashion [6,7,8]. Mertens et al. [6] proposed the fusion of images in a multi-scale manner based on pixel contrast, saturation, and well-exposedness to ease content inconsistency issues in the fused results. However, this often yields halo artifacts in its fusion results. In [7,8], the authors addressed the artifacts by applying modified guided image filtering to weight maps to eliminate halos around edges.

The abovementioned methods produce good results using a sequence of images exposed in a small interval of different exposure values (EV). Thanks to advanced sensor technology, a camera with Binned Multiplexed Exposure High-Dynamic-Range (BME-HDR) or Spatially Multiplexed Exposure High-Dynamic-Range (SME-HDR) technology can simultaneously capture an image pair with short- and long-exposure image sensors. The captured pair has only a negligible difference, possibly caused by local motion blur between them. The existing MEF methods may not work well with two exposure images, since none of the inputs may have well-exposed contents. In addition, weighted-sum fusion based on well-exposedness may not be able to deal with highlighted regions of a short-exposure image that are darker than dark parts in a long-exposure image, resulting in the method ignoring contents in the short-exposure image. Yang et al. [9] proposed the production of an intermediate virtual image with a medium exposure based on an image pair with two exposures to help generate better fusion results. Nevertheless, it does not work in situations where highlighted regions of both input LDR images are not well exposed.

In recent years, deep convolutional neural networks (CNNs) have gained tremendous success in low-level image processing works. In MEF, CNN-based methods [10,11] can better learn features from input multiple-exposure images and fuse them into a nice image. However, the fused images often lack image details [12], since spatial information may be lost when features pass through deep layers. Xu et al. [13] proposed a unified unsupervised image fusion network trained based on the importance and information carried by the two input images to generate fusion results. However, these learning-based methods can only produce a fused image based on the two input images’ interpolation. They cannot deal with cases where both of the input images do not have highlighted regions/contents.

This paper presents a two-exposure fusion framework that generates a more helpful intermediate virtual image for fusion using the proposed Optimized Adaptive Gamma Correction (OAGC). The virtual image has better contrast, saturation, and well-exposedness, and it is not restricted to being an interpolated version of the two input images. Fusing the input images with their virtual image processed by OAGC works well even though both inputs have no well-exposed contents or regions. Figure 1b shows an example where the proposed framework can still generate a good fusion result for when both of the input images lack highlighted regions (Figure 1a). Our primary contributions are three-fold:

- Our image fusion framework adopting the proposed OAGC can produce better fusion results for two input images with various exposure ratios, even when both of the input images lack well-exposed regions.
- The proposed framework with OAGC can also adapt to single-image enhancement.
- We conduct an extensive experiment using a public multi-exposure dataset [14] to demonstrate that the proposed fusion framework performs favorably against the state-of-the-art image fusion methods.

## 2. Related Work

MEF-based methods produce fusion results using a weighted combination of the input images based on each pixel’s “well-exposedness”. In [15], fusion weight maps were calculated based on the correlation-based match and salience measures of the input images. With the weight maps, one can fuse the input images into one by using the gradient pyramid.

Mertens et al. [6] constructed fusion weight maps based on contrast, saturation, and exposedness of the input images. Differently from [15], the fusion was performed with the Gaussian and Laplacian pyramids. The problem was that using the smoothed weight maps in fusion often causes halo artifacts, especially around the edges. The method proposed in [7] addressed this issue by applying an edge-preserving filter (weighted guided image filtering [16]) to fusion weight maps. Kou et al. [8] further proposed an edge-preserving gradient-domain guided image filter (GGIF) to avoid generating halo artifacts in the fused image. To extract image details, Li et al. [7] proposed a weighted structure tensor to manipulate details presented in a fused image. In general, MEF-based methods can generate decent fusion results.

General MEF algorithms [6,8] that require a sequence of images with different exposure ratios as the inputs may not work with only two input images. Yang et al. [9] proposed the use of the MEF algorithm for two-exposure-ratio image fusion, where an intermediate virtual image with a medium exposure is generated to help produce a better fusion result. However, the virtual image’s intensity and exposedness are bounded by the two input images, which often fails to work for cases where two images are both underexposed and overexposed. Yang’s method [9] can only generate both the intermediate and fusion results with approximate medium exposure between its two input images. The problem is that medium exposure between the inputs may still be under- or overexposure. Image fusion will not improve visual quality. We will discuss this issue more in the next section.

In the following paragraphs, we introduce the techniques adopted in the work of Yang et al., including the generation of the virtual image and fusion weights and the multi-scale image fusion. Before continuing, we define several notations that are used here. Let $I\in R^{M\times N\times 3}$ be a color image. We denote $I^{\left(c\right)}$ as the color channel *c*, where c∈{R,G,B} stand for the red, green, and blue channels. $I\left(m,n\right)$ represents the pixel located at $\left(m,n\right)$, where 0≤m<M and 0≤n<N. *M* and *N* are the image width and height. Let Y be the luminance component or the grayscale version of I. Note that the values of images in this paper are normalized to $\left[0,1\right]$.

### 2.1. Quality Measures and Fusion Weight Maps

In HDR imaging, an image taken at a certain exposure may contain underexposed or overexposed regions, which are less informative and should be assigned fewer weights in multi-exposure fusion. The input’s contrast, saturation, and well-exposedness determine a pixel’s weight at $\left(m,n\right)$ [6]. The contrast of a pixel, denoted by $C\left(m,n\right)$, is obtained by applying a 3×3 Laplacian filter to a grayscale version of the image:

(1) $$C\left(m,n\right)\;=\;\left|4Y\left(m,n\right)\;-\;Y\left(m-1,n\right)\right.\;-\;\left.Y\left(m,n-1\right)\;-\;Y\left(m+1,n\right)\;-\;Y\left(m,n+1\right)\right|.$$

Let $C=\;\left(\begin{array}{c} C\left(m,n\right) \end{array}\right)$ be the map of the contrast of I; therefore,

$$C=\;\left|4Y-Y_{l}-Y_{r}-Y_{u}-Y_{d}\right|,$$

where $Y_{l}$, $Y_{r}$, $Y_{u}$, and $Y_{d}$ are obtained from $I_{l}$, $I_{r}$, $I_{u}$, and $I_{d}$; i.e., shifting I one pixel left, right, up, and down, respectively. The saturation of the pixel, denoted by $S\left(m,n\right)$, is obtained by computing the standard deviation across the red, green, and blue channels:

(2) $$S\left(m,n\right)\;=\sqrt{\frac{1}{3}\sum_{c\in \left\{R,G,B\right\}}{\left[I^{\left(c\right)}\left(m,n\right)\;-\;\bar{I}\left(m,n\right)\right]}^{2}},$$

where

$$\bar{I}\left(m,n\right)\;=\frac{1}{3}\sum_{c\in \left\{R,G,B\right\}}I^{\left(c\right)}\left(m,n\right).$$

The well-exposedness of the pixel, $E\left(m,n\right)$, is defined as:

(3) $$E\left(m,n\right)\;=\exp\left[-\frac{1}{2\sigma^{2}}\sum_{c\in \left\{R,G,B\right\}}{\left(I^{\left(c\right)}\left(m,n\right)\;-\;\xi \right)}^{2}\right],$$

where σ=0.2 and ξ=0.5. Essentially, *E* is a normal distribution centered at 0.5 with a standard deviation of 0.2. The maps of saturation and well-exposedness of I can, respectively, be represented as $S=\;\left(S\left(m,n\right)\right)$ and $E=\;\left(E\left(m,n\right)\right)$. Next, the weight of the pixel for fusion is computed using:

(4) $$\tilde{W}\left(m,n\right)\;=C^{\omega_{c}}\left(m,n\right)S^{\omega_{s}}\left(m,n\right)E^{\omega_{e}}\left(m,n\right),$$

where $\omega_{c}$, $\omega_{s}$, and $\omega_{e}$ can be adjusted to emphasize or ignore one or more measures. Considering a set of *P* images $I_{1},\ldots ,I_{P}$ for image fusion, the weight of this pixel in the $p_{th}$ image is normalized by the sum of the weights across all the images at the same pixel:

(5) $$W_{p}\left(m,n\right)\;=\frac{{\tilde{W}}_{p}\left(m,n\right)}{\sum_{p^{\prime }=1}^{P}{\tilde{W}}_{p^{\prime }}\left(m,n\right)}.$$

The weight map of the image $I_{p}$ is represented as $W_{p}=\;\left(W_{p}\left(m,n\right)\right)$.

### 2.2. Multi-Scale Fusion

In the MEF algorithm [6], a fusion image, $\hat{I}$, is obtained through multi-scale image fusion based on the standard Gaussian and Laplacian pyramids. For each input image $I_{p}$ in the set of ${\left\{I_{p}\right\}}_{p=1}^{P}$, the Laplacian pyramid, $L^{\left(l\right)}\left\{I_{p}\right\}$, and the Gaussian pyramid of its weight map, $G^{\left(l\right)}\left\{W_{p}\right\}$, in the $l_{th}$ level are constructed by applying the Gaussian pyramid generation [17]. In this level, the overall Laplacian pyramid is collapsed by performing weighted averaging on the Laplacian pyramids from all of the input images in the set:

(6) $$L^{\left(l\right)}\{\hat{I}\}=\sum_{p=1}^{P}\left(G^{\left(l\right)}\left\{W_{p}\right\}⊙L^{\left(l\right)}\left\{I_{p}\right\}\right),$$

where ⊙ denotes element-wise multiplication. Finally, the fusion image, $\hat{I}$, is reconstructed by collapsing the Laplacian pyramids $L^{\left(l\right)}\{\hat{I}\}$.

Applying edge-preserving filtering to preserve edges in the weight maps before averaging the Laplacian pyramids in Equation (6) can reduce halo artifacts in fused images. In [9], the GGIF [18] was adopted to smooth the weight maps $W_{p}$ and to preserve the significant change as well. Let $\Omega_{\rho }\left(m_{0},n_{0}\right)$ be the square local patch with a radius of ρ centered at $\left(m_{0},n_{0}\right)$, and let $\left(m,n\right)$ be a pixel in the patch. In $\Omega_{\rho }\left(m_{0},n_{0}\right)$, the weight map in the $l_{th}$ level of the $p_{th}$ image, $W_{p}^{\left(l\right)}\left(m,n\right)$, is the linear transform of the luminance component, $Y_{p}^{\left(l\right)}\left(m,n\right)$:

(7) $$Y_{p}^{\left(l\right)}\left(m,n\right)\;=a_{p,\left(m_{0},n_{0}\right)}^{\left(l\right)}W_{p}^{\left(l\right)}\left(m,n\right)\;+\;b_{p,\left(m_{0},n_{0}\right)},$$

where $a_{p,\left(m_{0},n_{0}\right)}^{\left(l\right)}$ and $b_{p,\left(m_{0},n_{0}\right)}^{\left(l\right)}$ are the coefficients and are assumed to be constant in $\Omega_{\rho }\left(m_{0},n_{0}\right)$. $a_{p,\left(m_{0},n_{0}\right)}^{\left(l\right)}$ and $b_{p,\left(m_{0},n_{0}\right)}^{\left(l\right)}$ can be obtained by minimizing the objective function:

(8) $$\Lambda_{GIF}=\sum_{\begin{array}{c} m,n\in \\ \Omega_{\rho }\left(m_{0},n_{0}\right) \end{array}}\left[\left(a_{p,\left(m_{0},n_{0}\right)}^{\left(l\right)}W_{p}^{\left(l\right)}\left(m,n\right)\;+\;b_{p,\left(m_{0},n_{0}\right)}^{\left(l\right)}\;-\;\right.\right.{\left.Y_{p}^{\left(l\right)}\left(m,n\right)\right)}^{2}-\left.\epsilon {\left(a_{p,\left(m_{0},n_{0}\right)}^{\left(l\right)}\right)}^{2}\right],$$

where ϵ is a constant for regularization. The variance of the intensities within this local patch, $\sigma_{Y_{p}^{\left(l\right)},\Omega }^{2}$, is computed when solving for the coefficients in Equation (8).

In GGIF, a 3×3 local window, Ψ, is applied to the pixels within $\Omega_{\rho }\left(m_{0},n_{0}\right)$ for capturing the structure within $\Omega_{\rho }\left(m_{0},n_{0}\right)$ by computing the variance within Ψ, $\sigma_{Y_{p}^{\left(l\right)},\Psi }^{2}$ [18]. This local window makes GGIF a content-adaptive filter; thus, GGIF produces fewer halos and better preserves the edge than the GIF. In GGIF, the regularization term is designed to yield:

(9) $$\Lambda_{GG}=\sum_{\begin{array}{c} m,n\in \\ \Omega_{\rho }\left(m_{0},n_{0}\right) \end{array}}\left[\left(a_{p,\left(m_{0},n_{0}\right)}^{\left(l\right)}W_{p}^{\left(l\right)}\left(m,n\right)\;+\;b_{p,\left(m_{0},n_{0}\right)}^{\left(l\right)}\right.\right.{\left.\;-\;Y_{p}^{\left(l\right)}\left(m,n\right)\right)}^{2}-\left.\lambda \frac{{\left(a_{p,\left(m_{0},n_{0}\right)}^{\left(l\right)}-\zeta_{\left(m_{0},n_{0}\right)}\right)}^{2}}{\Gamma_{Y_{k}}\left(m_{0},n_{0}\right)}\right],$$

where $\Gamma_{Y_{k}}\left(m_{0},n_{0}\right)$ and $\zeta_{\left(m_{0},n_{0}\right)}$ are computed according to the product of $\sigma_{Y_{p}^{\left(l\right)},\Omega }$ and $\sigma_{Y_{p}^{\left(l\right)},\Psi }$ (the standard deviations of the pixels within $\Omega_{\rho }\left(m_{0},n_{0}\right)$ and Ψ), and λ is a constant for regularization. The filter coefficients $a_{p,\left(m_{0},n_{0}\right)}^{\left(l\right)}$ and $b_{p,\left(m_{0},n_{0}\right)}^{\left(l\right)}$ can solved by minimizing $\Lambda_{GG}$ in Equation (9).

The fused image $\hat{I}$ can be obtained by fusing the Laplacian pyramids of the input images taken at different exposures using the weight maps retrieved from the Gaussian pyramids, $G^{\left(l\right)}\left\{W_{p}\right\}$. Note that the weight maps are filtered using GGIF, as described in Equation (9), to preserve edges.

### 2.3. Virtual Image Generation

In [9], Yang et al. proposed the modification of two differently exposed images to have the same medium exposure using the intensity mapping function based on the cross-histogram between two images, called the comparagram (Ref. [19]), and fused them to produce an intermediate virtual image. Let $I_{1}$ and $I_{2}$ be the two input images and let $F_{12}$ and $F_{21}$ be the intensity mapping functions (IMFs) that map $I_{1}$ to $I_{2}$ and $I_{2}$ to $I_{1}$. Based on [19], the IMFs that map the two images to the same exposure, denoted as $F_{13}$ and $F_{23}$, are computed as

(10) $$F_{13}\left(z\right)\left(I_{i}\right)={\left(zF_{12}\left(z\right)\right)}^{0.5},\;F_{23}\left(z\right)={\left(zF_{21}\left(z\right)\right)}^{0.5},$$

where *z* is a pixel intensity. The two modified images with the same exposure are $\tilde{I_{i}}=F_{i3}\left(I_{i}\right)$, i=1,2. The desired virtual image $I_{v}$ is computed by fusing $\tilde{I_{1}}$ and $\tilde{I_{2}}$ using the weighting functions adopted in [9]. The two-exposure-fusion image in [9] is obtained by fusing $I_{1}$, $I_{2}$, and $I_{v}$ based on the MEF algorithm [8].

As described previously, Yang’s method often fails to produce a satisfying fusion result when the medium exposure between inputs is still under- or overexposure. The proposed method addresses this issue by improving the contrast, saturation, and well-exposedness for the intermediate virtual image to generate better fusion results under different input conditions.

## 3. Proposed Method

The algorithm in [9] can work for two images with a large difference between their exposure ratios. In this case, the intermediate virtual image with medium exposure helps bridge the dynamic range gap between the two inputs. Thus, it can improve the quality of the fusion result. However, if the two inputs’ exposure is under- or overexposure, the generated virtual image would not help fusion. Thus, the quality of the fused image is not improved much.

**Figure 2:**
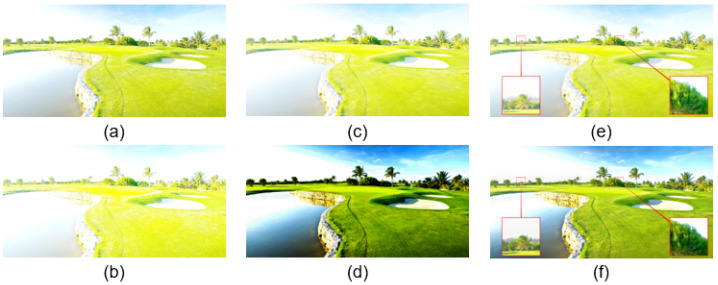
Comparison of intermediate virtual images. (**a**,**b**): Input images $I_{1}$ and $I_{2}$; (**c**,**d**) are the intermediate virtual images using [9] and our method; (**e**,**f**) are the fusion results using [9] and ours.

For example, to fuse Figure 2a,b, both of which look overexposed, the virtual image $I_{v}$ (Figure 2c) generated by [9] with medium exposure between the inputs is still overexposed and, thus, not helpful for the fusion result (Figure 2e). We propose Optimized Adaptive Gamma Correction (OAGC) to enhance the intermediate virtual image to have better contrast, saturation, and well-exposedness (Figure 2d) so that it can improve the fusion quality and produce a better result (Figure 2f).

In OAGC, we derive an optimal γ based on the input’s contrast, saturation, and well-exposedness by formulating an objective function based on these image quality metrics and apply it to the input image using gamma correction. Let $Y\left(m,n\right)$ be the luminance of a pixel. One can gamma-correct the image Y to alter its luminance through the power function as follows:

(11) $$Y_{\gamma }\left(m,n\right)\;=\eta Y{\left(m,n\right)}^{\gamma },$$

where $Y_{\gamma }$ is the corrected image, η and γ are positive scalars, and η is usually set to 1 [20]. Here, the notation $Y_{\gamma }$ in bold represents the entire image, while $Y_{\gamma }\left(m,n\right)$ stands for the pixel located at $\left(m,n\right)$. If γ<1, it stretches the contrast of shadow regions (pixel intensities less than the mid-tone of 0.5), and features in these regions become discernible, whereas if γ>1, it stretches the contrast of bright regions (intensities larger than 0.5), and features in the regions become perceptible. For γ=1, it is linear mapping.

To derive the optimal gamma, we design an objective function as follows:

(12) $$f\left(\gamma \right)\;=q_{1}{∥\hat{c}\left(\gamma \right)∥}^{2}+q_{2}{∥\hat{s}\left(\gamma \right)∥}^{2}+q_{3}{∥\hat{e}\left(\gamma \right)∥}^{2}+\delta {∥\hat{r}\left(\gamma \right)∥}^{2},$$

where $\hat{c}\left(\gamma \right)\;=k_{c}1-\mathrm{vec}\left(C_{\gamma }\right)$, $\hat{s}\left(\gamma \right)\;=k_{s}1-\mathrm{vec}\left(S_{\gamma }\right)$, $\hat{e}\left(\gamma \right)\;=k_{e}1-\mathrm{vec}\left(E_{\gamma }\right)$, and $\hat{r}\left(\gamma \right)\;=\mathrm{vec}\left(I_{v}-I_{\gamma }\right)$, where $C_{\gamma }$, $S_{\gamma }$, and $E_{\gamma }$ are the maps of quality measures computed based on the gamma-corrected version of the input image, denoted as $I_{\gamma }$. Here, the virtual image $I_{v}$ is used as the input, which is $I_{\gamma }:=I_{v}^{\gamma }$. We set $k_{c}$, $k_{s}$, and $k_{e}$ to 4, 0.5, and 1 according to the upper bounds of the corresponding quality measures (contrast, saturation, and well-exposednesse; refer to the Appendix A for the derivation). The term with $\hat{r}\left(\gamma \right)$ in the objective function prevents the corrected image from deviating the input too much. Hence, minimizing the objective function $f\left(\gamma \right)$ is to maximize all three quality measures: the contrast, saturation, and well-exposedness. $q_{1}$, $q_{2}$, and $q_{3}$ are the weighting factors for the contributions from different quality measures (independent from $\omega_{c}$, $\omega_{s}$, and $\omega_{e}$ in Equation (4) and are all set to $\frac{1}{3}$. δ is a small, fixed scalar and is set to 0.1 in the present study. 1 is the vector of 1s, $\mathrm{vec}\left(\cdot \right)$ is the vectorization of a matrix, and $∥\cdot ∥$ represents the 2-norm of a vector. The regularization term is added to avoid possible color distortion caused by gamma correction.

The optimal gamma, $\gamma^{*}$, which aims to increase contrast, saturation, and well-exposedness simultaneously, can be obtained by minimizing the optimization function $f\left(\gamma \right)$:

(13) $$\gamma^{*}=\arg\min_{\gamma }f\left(\gamma \right).$$

Since there is no closed-form solution for Equation (13), we apply the gradient descent to iteratively approximate it:

(14) $$\begin{array}{cc} \gamma^{\left(k+1\right)}=\gamma^{\left(k\right)}\;+ & \alpha^{\left(k\right)}\frac{d}{d\gamma }f\left(\gamma \right)\\ =\gamma^{\left(k\right)}\;+ & \alpha^{\left(k\right)}\left[q_{1}{\left(\frac{d}{d\gamma }\hat{c}\left(\gamma \right)\right)}^{T}\hat{c}\left(\gamma \right)\right.\;+\;\left.q_{2}{\left(\frac{d}{d\gamma }\hat{s}\left(\gamma \right)\right)}^{T}\hat{s}\left(\gamma \right)\right.\\ \;+ & \left.q_{3}{\left(\frac{d}{d\gamma }\hat{e}\left(\gamma \right)\right)}^{T}\hat{e}\left(\gamma \right)\right.\;+\;\left.\delta {\left(\frac{d}{d\gamma }\hat{r}\left(\gamma \right)\right)}^{T}\hat{r}\left(\gamma \right)\right], \end{array}$$

where

(15) $$\begin{array}{cc} \frac{d}{d\gamma }\hat{c}\left(\gamma \right) & =-\mathrm{sgn}\left[\sum_{c\in \left\{R,G,B\right\}}t^{\left(c\right)}\left(4i_{v}^{{\left(c\right)}^{\gamma }}\right.\right.-\left.\left.i_{v,l}^{{\left(c\right)}^{\gamma }}-i_{v,r}^{{\left(c\right)}^{\gamma }}-i_{v,u}^{{\left(c\right)}^{\gamma }}-i_{v,d}^{{\left(c\right)}^{\gamma }}\right)\right]\\ \; & \times \left[\sum_{c\in \left\{R,G,B\right\}}t^{\left(c\right)}\left(4i_{v}^{{\left(c\right)}^{\gamma }}⊙\log i_{v}\right.\right.-i_{v,l}^{{\left(c\right)}^{\gamma }}⊙\log i_{v,l}-i_{v,r}^{{\left(c\right)}^{\gamma }}⊙\log i_{v,r}\\ \; & \;\left.\left.-i_{v,u}^{{\left(c\right)}^{\gamma }}⊙\log i_{v,u}-i_{v,d}^{{\left(c\right)}^{\gamma }}⊙\log i_{v,d}\right)\right], \end{array}$$

with $i_{v,l}$, $i_{v,r}$, $i_{v,u}$, and $i_{v,d}$ being the vectorization of $I_{v,l}$, $I_{v,r}$, $I_{v,u}$, and $I_{v,d}$, as well as $t^{\left(R\right)}$, $t^{\left(G\right)}$, and $t^{\left(B\right)}$ being 0.299, 0.587, and 0.114 respectively.

(16) $$\begin{array}{cc} \frac{d}{d\gamma }\hat{s}\left(\gamma \right) & =-\frac{1}{9}\left[2\sum_{c\in \left\{R,G,B\right\}}\left(i_{v}^{{\left(c\right)}^{\left(2\gamma \right)}}⊙\log i_{v}^{\left(c\right)}\right)\right.\\ & -\;i_{v}^{{\left(R\right)}^{\gamma }}⊙i_{v}^{{\left(G\right)}^{\gamma }}⊙\log\left(i_{v}^{{\left(R\right)}^{\gamma }}+i_{v}^{{\left(G\right)}^{\gamma }}\right)\\ & -\;i_{v}^{{\left(G\right)}^{\gamma }}⊙i_{v}^{{\left(B\right)}^{\gamma }}⊙\log\left(i_{v}^{{\left(G\right)}^{\gamma }}+i_{v}^{{\left(B\right)}^{\gamma }}\right)\\ & -\;\left.i_{v}^{{\left(B\right)}^{\gamma }}⊙i_{v}^{{\left(R\right)}^{\gamma }}⊙\log\left(i_{v}^{{\left(B\right)}^{\gamma }}+i_{v}^{{\left(R\right)}^{\gamma }}\right)\right]⊘s_{\gamma }, \end{array}$$

with ⊘ being the element-wise division,

(17) $$\frac{d}{d\gamma }\hat{e}\left(\gamma \right)=\frac{e_{\gamma }}{2\sigma^{2}}⊙\sum_{c\in \left\{R,G,B\right\}}\left[\left(2i_{v}^{{\left(c\right)}^{2\gamma }}-i_{v}^{{\left(c\right)}^{\gamma }}\right)⊙\log i_{v}^{\left(c\right)}\right],$$

(18) $$\frac{d}{d\gamma }\hat{r}\left(\gamma \right)=\sum_{c\in \left\{R,G,B\right\}}\left[\left(i_{v}^{{\left(c\right)}^{\left(2\gamma \right)}}-i_{v}^{{\left(c\right)}^{\left(\gamma +1\right)}}\right)⊙\log i_{v}^{\left(c\right)}\right],$$

and $\alpha^{\left(k\right)}$ is the adjustable learning rate.

**Figure 3:**
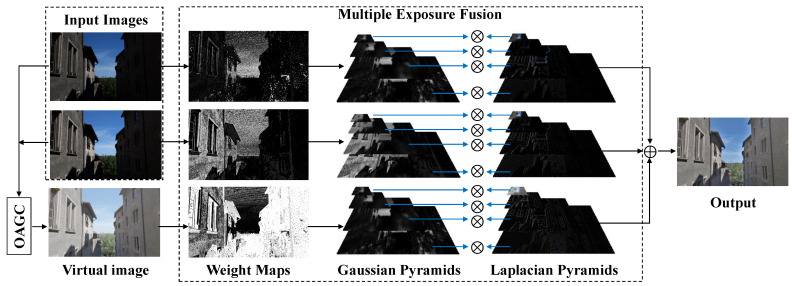
Flowchart of the proposed method. Note that the images of Gaussian and Laplacian pyramids are contrast-enhanced for display.

Figure 3 shows the flowchart of the presented two-exposure image fusion framework, where the two inputs are taken in the same scene at different exposure ratios. The virtual image is first generated using the intensity mapping function [9]. Next, we solve Equation (12) to find the optimal gamma value $\gamma^{*}$ for the virtual image, which enhances the contrast, saturation, and well-exposedness of $I_{v}$. The final fused image, $\hat{I}$, is obtained by applying the MEF algorithm [8,9] to the fusion of two input images and $I_{\gamma }$.

## 4. Experimental Results

In the experiment, we compared the proposed method against state-of-the-art image fusion methods, which included Kou’s method [8], DeepFuse [10], Yang’s method [9], and U2Fusion [13]. We adopted the SICE datasest [14] and collected 116 image pairs that consisted of various scenes to evaluate the performance. The presented algorithm was implemented using MATLAB R2019b on a MacBook Pro with an Intel i5 dual-core processor at 2.7 GHz and 8 GB 1867 MHz DDR3 RAM. We present a performance evaluation with a qualitative visual comparison and a quantitative objective assessment in the following.

### 4.1. Qualitative Assessment

**Figure 4:**
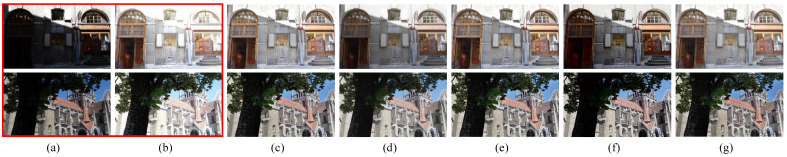
Comparison of the results obtained using different fusion methods with an underexposed and an overexposed input. (**a**,**b**) show the input images squared in red. The fusion results were obtained using (**c**) Kou’s method [8], (**d**) DeepFuse [10], (**e**) Yang’s method [9], (**f**) U2Fusion [13], and (**g**) the proposed method.

We compared different fusion results under various input conditions. First, Figure 4 shows the fusion results of using the compared image fusion algorithms [8,9,10,13] and our presented framework. As can be seen, one input image is underexposed, and the other is overexposed in the two cases, where the fusion results should have middle exposure between the two inputs. All of the compared methods worked fine in such cases, although U2fusion’s [13] fusion results were a little darker than the others’ results.

**Figure 5:**
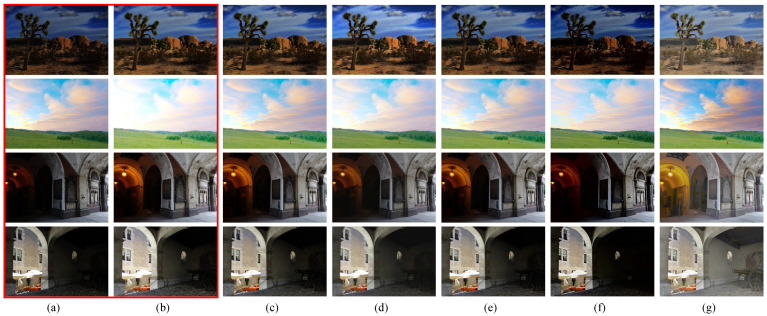
Comparisons of the fusion results using different algorithms, where the two input images had smaller exposure differences. (**a**,**b**) show the input images squared in red. The fusion results were obtained using (**c**) Kou’s method [8], (**d**) DeepFuse [10], (**e**) Yang’s method [9], (**f**) U2Fusion [13], and (**g**) the proposed method.

Figure 5 shows image fusion cases where the difference between the two input images’ EVs was not large. Thus, fusion methods that can only produce results with medium exposure between the inputs do not work. As shown, all of the compared methods except for the proposed framework output fusion results similar to the input images, and were thus unable to reveal more details than the inputs. In contrast, the proposed framework produced an intermediate virtual image enhanced by OAGC with additional well-exposed highlighted contents and generated better fusion results. Therefore, we can further improve the overall image visibility by revealing details in regions that are too dark or bright.

### 4.2. Quantitative Assessment

Objectively, we compare the performance of our presented framework against other image fusion methods using five benchmark metrics: the Naturalness Image Quality Evaluator (NIQE) [21], Blind/Referenceless Image Spatial Quality Evaluator (BRISQUE) [22], No-Reference Image Quality Assessment (NR-IQA) [23], and discrete entropy (DE) [24]. The NIQE [21] is a no-reference image quality metric that is trained on pristine images without subjective scores from humans. Therefore, it can measure image quality degradation if any distortions exist, but is correlated little with human perceptuality. A smaller value means a better quality. BRISQUE [22] is a natural scene statistics-based distortion-generic no-reference image quality assessment model that is trained on images with known distortions and subjective quality scores. It can evaluate losses of naturalness of an image caused by possible distortions. A BRISQUE value ranges between 0 and 100. A smaller value means worse visual quality. NR-IQA [23] is another no-reference image quality metric for HDR images that is constructed using deep CNNs while considering image saliency, and it constructs deep CNNs to extract quality features across the HDR and LDR domains. DE [24] can represent the information contained in an information source, i.e., if an image has higher entropy, it contains more information. Consequently, it is often used to measure the richness of image details. It is defined as:

(19) $$\mathrm{DE}\left(I\right)=-\sum_{l=0}^{L-1}p_{I}\left(l\right)\log\left(p_{I}\left(l\right)\right),$$

where *I* is a grayscale image, *L* represents the largest pixel intensity value, and $p_{I}\left(l\right)$ is the probability density function of a given grayscale intensity *l*.

Table 1 shows the quantitative performance of different fusion methods, where the scores are averaged over all of the test images. As can be seen, the results demonstrate that the presented framework achieved the best scores in all four categories, meaning that our fusion results looked natural with the fewest distortions (having the lowest NIQE [21] and lowest BRISQUE values [22]). In assessing the HDR image quality (NR-IQA [23]), our method performed favorably against other fusion methods. Our method could also preserve the most image details in the fusion results (largest DE value [24]).

### 4.3. Extension to Single Image Enhancement

As stated previously, the proposed framework works well for two-exposure image fusion in cases where the difference between the two input images’ EVs varies. In recent years, fusion-based single image enhancement methods have attracted much attention [14]. We can also extend our framework to single image enhancement by applying OAGC to the input image, I, to yield a quality-improved image $I_{\gamma }$. Then, both I and $I_{\gamma }$ are fused to obtain an enhancement result.

**Figure 6:**
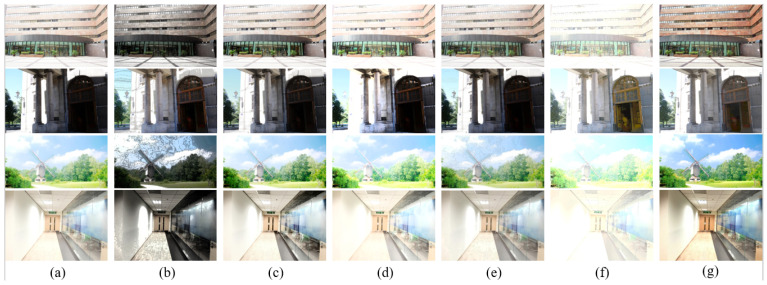
Comparison of single image enhancement results using different algorithms. (**a**) Input image; the enhanced results obtained using (**b**) HE [25], (**c**) CVC [26], (**d**) AGCWD [27], (**e**) EPMP [28], (**f**) SICE [14], and (**g**) the proposed method (fusing I and $I_{\gamma }$).

Figure 6 compares the results obtained using various single image enhancement methods, including global histogram equalization (HE) [25], CVC [26], AGCWD [27], EPMP [28], SICE [14], and the proposed method. As shown, the conventional HE tended to over-enhance/introduce noise to the processed images (Figure 6b), since the input images had over- and underexposed regions. SICE [14] only performed well for the second row of Figure 6, where the input image was underexposed. For the other cases, it tended to overexpose the input images. The other methods [26,27,28] could only enhance the contrast of the input images, while the proposed framework not only did that, but also revealed unseen details from the input and increased the color vividness (Figure 6g).

### 4.4. Analysis of OAGC

**Figure 7:**
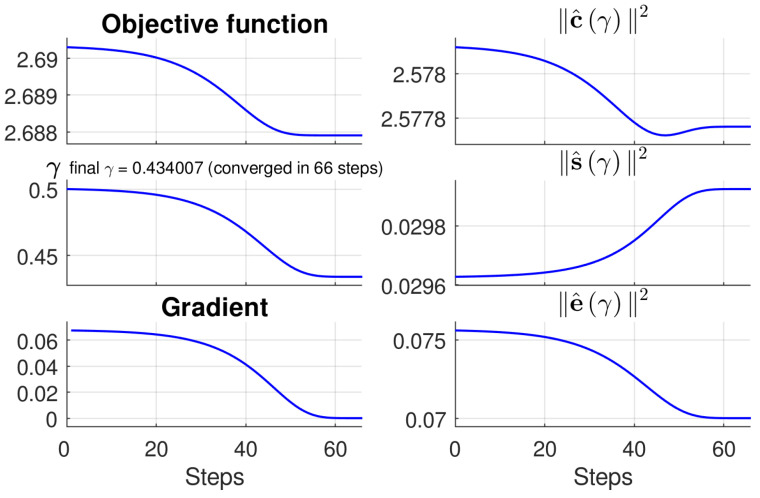
The progressive process of attaining $\gamma^{*}$ for an intermediate virtual image using Equation (12) (taking $I_{v}$ in the top row of Figure 5 as an example).

Convergence of Gradient Descent: To further analyze the process of attaining the target gamma value $\gamma^{*}$ in OAGC, we take the case in the top row of Figure 5 as an example to show the iterative steps of finding $\gamma^{*}$ for the intermediate virtual image $I_{v}$. Figure 7 shows that it takes about 66 steps for the objective function $f\left(\gamma \right)$ to converge with gradient descent, and it attains $\gamma^{*}=0.434$. The value of the objective function changes from the initial 2.690 to 2.688. As $\gamma =\gamma^{*}$, $\hat{e}\left(\gamma \right)$ reaches its minimum, and $\hat{c}\left(\gamma \right)$ is close to its minimum while $\hat{s}\left(\gamma \right)$ is at its maximum, indicating that contrast, saturation, and well-exposedness are all maximized. This also shows that solving the objective function strikes a balance among these three measures.

**Figure 8:**
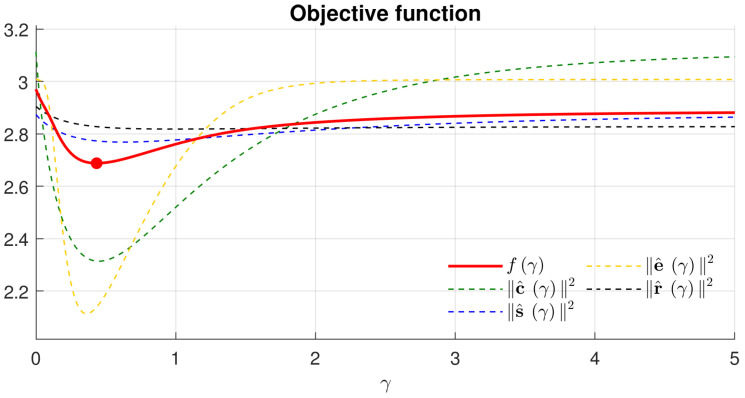
The grid search of $\gamma^{*}$ (taking $I_{v}$ in the top row of Figure 5 as an example).

To further attest to the effectiveness of OAGC, Figure 8 shows the trend of values of the objective function and its quality measure terms using the grid-search method on γ. As seen, the minimum of the objective function is 2.688 when γ=0.434, consistently with the $\gamma^{*}$ obtained using gradient descent.

**Figure 9:**
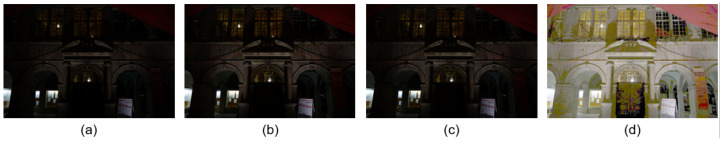
Limitation of OAGC. (**a**,**b**) Input images. (**c**) Virtual image $I_{v}$ using [9]; (**d**) virtual image processed using OAGC $I_{\gamma }$.

Limitation of OAGC: Using OAGC, we can attain a gamma coefficient $\gamma^{*}$ from the input image by optimizing the objective function in Equation (12), and we can then apply gamma correction to the input to generate a corrected image whose contrast, saturation, and well-exposedness are improved. However, if both of the input images have no content at all for the same regions due to extremely low or high exposure, even OAGC cannot help generate or restore those regions from nothing. Figure 9 shows a failure case of OAGC, where both of the input images are very underexposed and bear little content. Figure 9c shows the intermediate virtual images obtained using the intensity mapping algorithm described in [9], which is similar to the interpolation of the inputs, and the result is still very dark and lacks content. After applying OAGC to it ($\gamma^{*}=0.2015$), the processed virtual image (Figure 9d) presents more details than before. Still, it inevitably has noise in some regions (such as the door and the banner on the façade).

## 5. Conclusions

This paper presented a two-exposure image fusion framework that utilizes the proposed OAGC to bring out additional well-exposed contents from an intermediate virtual image derived from the two inputs. It can work better for the input images with various combinations of exposure ratios and can produce more well-exposed fusion results. In addition, the proposed framework with OAGC can easily adapt to single image enhancement. The experimental results have demonstrated that the proposed method performs favorably against the state-of-the-art image fusion methods.

## Figures and Tables

**Table 1:** Quantitative comparisons of different fusion methods. The scores are averaged over all of the test images. The values in bold represent the best scores.

	NIQE ↓	BRISQE ↓	NR-IQA ↑	DE ↑
Kou [8]	2.7866	22.7140	43.6202	6.9171
DeepFuse [10]	2.9555	31.5365	32.9696	6.9564
Yang [9]	2.7895	23.0660	43.5312	6.8617
U2Fusion [13]	3.421	35.5939	34.5449	6.4195
Proposed	**2.7339**	**21.7120**	**43.8968**	**6.9814**

## Appendix A. Upper Bounds of the Quality Measures

Since the luminance of the pixel is $Y\left(m,n\right)\;\in \left[0,1\right]$, the upper bound of its contrast value $C\left(m,n\right)$ is:

(A1) $$C\left(m,n\right)\;=\;\left|4Y\left(m,n\right)\;-\;Y\left(m-1,n\right)\right.\;-\;\left.Y\left(m,n-1\right)\;-\;Y\left(m+1,n\right)\;-\;Y\left(m,n+1\right)\right|\;\leq \;4.$$

For exposedness, $E\left(m,n\right)$ is shown as:

(A2) $$E\left(m,n\right)\;=\exp\left[-\frac{1}{2\sigma^{2}}\sum_{c\in \left\{R,G,B\right\}}{\left(I^{\left(c\right)}\left(m,n\right)\;-\;\xi \right)}^{2}\right]\leq 1,$$

where $E\left(m,n\right)\;=1$ when $I^{\left(c\right)}\left(m,n\right)\;=\xi$.

The saturation $S\left(m,n\right)$ is defined as in Equation (2). Let $\bar{I}\left(m,n\right)$ be the mean of all the channels of this pixel; i.e.,

$$\bar{I}\left(m,n\right)\;=\frac{1}{3}\sum_{c\in \left\{R,G,B\right\}}I^{\left(c\right)}\left(m,n\right).$$

The upper bound of $S\left(m,n\right)$ can be obtained from:

$$\begin{array}{cc} S^{2}\left(m,n\right) & =\frac{1}{3}\sum_{c\in \left\{R,G,B\right\}}{\left[I^{\left(c\right)}\left(m,n\right)\;-\;\bar{I}\left(m,n\right)\right]}^{2}\\ \; & =\frac{1}{3}\left[\sum_{c\in \left\{R,G,B\right\}}I^{\left(c\right)}{\left(m,n\right)}^{2}\right.-\left.2\bar{I}\left(m,n\right)\sum_{c\in \left\{R,G,B\right\}}I^{\left(c\right)}\left(m,n\right)\;+\;3\bar{I}{\left(m,n\right)}^{2}\right]\\ \; & =\frac{1}{3}\left[\sum_{c\in \left\{R,G,B\right\}}I^{\left(c\right)}{\left(m,n\right)}^{2}-3\bar{I}{\left(m,n\right)}^{2}\right]. \end{array}$$

Because $I^{\left(c\right)}\left(m,n\right)\;\in \;\left[0,1\right]$,

$$\sum_{c\in \left\{R,G,B\right\}}I^{\left(c\right)}{\left(m,n\right)}^{2}\leq \sum_{c\in \left\{R,G,B\right\}}I^{\left(c\right)}\left(m,n\right)\;=3\bar{I}\left(m,n\right).$$

This indicates that

$$\begin{array}{cc} S{\left(m,n\right)}^{2} & \leq \bar{I}\left(m,n\right)\;-\;\bar{I}{\left(m,n\right)}^{2}\\ \; & =-{\left(\bar{I}\left(m,n\right)\;-\;0.5\right)}^{2}+0.25\leq 0.25. \end{array}$$

Therefore, the upper bound of $S\left(m,n\right)\;=0.5$.
